# Designing Solutions for the Retirement System – In Search of Balance between Economy and Health

**DOI:** 10.3389/fpubh.2016.00184

**Published:** 2016-08-31

**Authors:** Piotr Romaniuk, Katarzyna Brukało

**Affiliations:** ^1^Department of Health Policy, School of Public Health in Bytom, Medical University of Silesia in Katowice, Bytom, Poland

**Keywords:** social security, social insurance, health status, occupational health, healthy life years

## Abstract

Social security system currently faces a number of difficulties arising of changes in the demographic structure of societies, like the decrease in fertility, lengthening of life expectancy, and unfavorable change in the proportion of the population receiving retirement benefits to the population in working age. In result, social security systems are being subjected to transition aimed at securing their financial stability, part of which is a tendency to rise the retirement age and eliminate all the incentives to prematurely exit the labor market. On the other hand, this process of transition, as observed in Poland, is being driven mainly by political processes and due to economic reasons, while lacking public health evidence. This raises a danger that in final result the financial savings will be illusory only and that the final configuration of the system will be inconsistent with the actual social needs of the population and will not efficiently protect against the social risks. In this article, we present arguments for using the Healthy Life Years indicator in analyses relating to the performance of social security systems. The indicator may help to reflect differences in health status of different professional groups and adjust system’s solutions to conditions characterizing these groups, in terms of both risk protection and prevention.

## Introduction

Long life is perceived, nowadays, as a supreme, highly desired value. Constantly expanding life expectancy is being taken as a proof of effectiveness of public, social, and health policy systems, often as one of the most basic factors to be taken into account when assessing health system efficiency. Nonetheless, long life has real value only when it is accompanied by health and also professional productivity, maximized in nominal terms, and extended over time. The combination of life length and health is being depicted with indicators for the expected level of health (health expectancies). As such, it should remain in the center of interest of all sorts of institutions that deal with the national health policy at different decisional levels; including those which design plans of action to improve the population’s health and those which are responsible for financing health and other social services – to more accurately estimate the risk of sickness or disability (1).

The issue of healthy life expectancy measures has the potential to demonstrate its utility in two ways, in each case remaining in close connection with the attributes that characterize the various sectors of the national economy, from the perspective of their employees (2). On the one hand, any variation of the data for employees of various industries should be the basis for planning interventions to improve working conditions and, as a result, improve the health status of workers of these industries. On the other hand, the matter may play a key role in the design of solutions in the field of social insurance, such as disability insurance or retirement insurance – both in terms of risk assessment and the cost of the system, as well as in terms of the formal requirements constituting rights to benefits. This is all the more important, that these systems have in recent years faced with the rising difficulties violating their financial stability, which in turn becomes a prerequisite for the introduction of legislation to prolong the period of professional activity, or the age of entitlement to a similar type of benefits. As far as we are often faced with justifying such measures by reference to data showing lengthening life expectancy, it seems that the question of healthy life expectancy is ignored in discussions on this kind of issues. In light of the above, the purpose of this article is to demonstrate the desirability of using the indicator of healthy life years in the design of solutions in the field of social security, based on the data characterizing Polish system.

## The Retirement System as a Protection from Risk of Infirm Old Age

According to the classic assumptions of public social security systems, such systems should be used to finance the basic needs of people during their disability to acquire income by themselves, including the infirm old age. In the days of the formation of public social security systems, in reality, very few people reached such an age, because, at that time, the average LE was about 45 years, while the retirement age – between 60 and 70 years. Thus, according to the assumption, people who reached retirement age were actually unable to secure livelihoods by themselves (3).

However, due to demographic changes and the evolution of the social security system, working people have acquired many rights and privileges, which led to a situation where the labor market is being left by those, who are not yet at the infirm old age, and, in many cases, depending on the sector of economy they used to work in, might function without major difficulty (3). It can, therefore, be concluded that, as a result of such changes in the social security system, it has become rather a tool for the allocation of income in the life cycle, not the way to financially secure infirm old age (2). In view of the foregoing, it seems reasonable to question whether it is appropriate to take a part of the income generated by the working part of a population, and transfer it to people who, by economic considerations, might still participate in its generation (3). Due to lack of evidence of a medical or biological nature, to make this kind of transfers, together with the evolution of social security systems, in place of the traditional justification for the existence of retirement pension insurance, it started rather to be used the criterion of merit, which leads to allow workers a proper rest after a period of professional activity, as a kind of reward for their contribution to the development of the national economy, but also to the maintenance of the social security system (4).

## Health as a Key Determinant of Economic Development

Demographic phenomena negatively affecting financial stability of social security systems have turned again the discussion on the criteria of entitlement to retirement benefits into the issues related to health status and ability to continue professional activity. This, in turn, links the discussion to the question of health as a factor determining economic performance of a given society.

Undoubtedly, there is a link between health status of society as a whole, and individuals, and the economic performance of this society. This has been highlighted by the European Union in the proposal for establishing the “Health for Growth” program, the third multi-annual program of the EU action in the field of health for years 2014–2020, which states: “Health problems are one of the major causes of absenteeism from work and early retirement. Keeping people healthy and active for longer has a positive impact on productivity and competitiveness. Increasing the number of healthy life years is a prerequisite if Europe is to succeed in employing 75% of 20–64 years old and avoiding early retirement due to illness. In addition, keeping people over 65 years of age healthy and active can impact on labor market participation and lead to potential important savings in health-care budgets” (5).

Thus, on the one hand, there is economic need for professional activation of older people, as indicated by the current demographic situation. The way to retain older people in the labor market is by limiting the incentives in a system of social security, which somehow allows early withdrawal from the labor market to people in nearly retirement age and lengthening of the retirement age, which has been done in many countries, including Poland. This type of reform is not widely supported or socially accepted, however, appears to be necessary. According to the judgment of the Polish Constitutional Court of 15 VII 2010: “The observed for many years change in cultural patterns and social conditions, in the form of roles of women and men in different spheres of life, the rise in popularity of partnership-based family model and women’s economic activity, create a new socioeconomic context for the determination of retirement age. Equalizing the retirement ages for men and women and, often, raising it also results from the adjustment of retirement systems to demographic phenomena: longer average LE, longer average life span for women than for men, improve in the health status of the population, decline in fertility, which causes a lack of replacement of generations. These phenomena, observed in all European countries, influence the process of aging, which could threaten the financial stability of social security systems and create problems on the labor market” (6).

On the other hand, the issue of the retirement system and incentives that are arising of its construction for earlier cessation of professional activity should not be discussed in separation from the problem of health risks associated with prolonged work experience under certain conditions. Some professions result in lowering the efficiency of the employee, negatively affect his performance, as well as – if to consider the issue from the perspective of the public social security system – generate additional costs related to the appearance of disability entailing the need for treatment, rehabilitation and benefits aimed at the maintenance of an adequate standard of living of a person (7).

## The Issue of Health as One of the Components in the Analyses of Professional Activity

As show the data of the Polish Ministry of Labor and Social Policy, good health coincides with increased economic activity. According to these data, those, who are retired, are clearly in worse health status: 10% of working men – pensioners declared own health status as bad or very bad, while among those, who were not entitled to receive retirement benefits, there was only 4% of such statements. In women, these rates were 11 and 3%, respectively. At the same time working retirees are healthier than retirees who are not active professionally – approximately 48% of the first group rate own health as good or very good, while among the unemployed this is not more than 35%. It can be concluded that the retirement and cessation of professional activity coincide with relatively poorer health. This, obviously, most probably, results from the fact, that those, who have better health status, are more willing to continue working, even when they already qualify to get retirement benefit. Their physical condition is not actually different from the one characterizing workers, who are still before retirement. As emphasized by the authors of the Ministry’s report: “relatively poorer health is conducive to retirement and cessation of activity” (8).

Health status was also one of the key determinants that are defined in the report “The exercise of care and other factors increasing professional activity in older-productive age.” The examined group in this study was women aged 50–65 years and men aged 55–70 years. It can be assumed that part of the research on the subjective assessment of health status was based on a modified GALA study. This study concluded that the deterioration of health is particularly pronounced for women aged 60–65 years, and for men aged 65–70 years, but in some cases even earlier. It appears, therefore, that keeping women in the labor market up to the age of 67 means the extension of the period of employment, while significantly deteriorates their health status, or limits in the possibility of performing basic life functions occur. At the same time, in the light of the foregoing, it should be emphasized that biological disability (feeling of limitations in the ability to perform basic life functions) occurs much less frequently than the legal disability (having a decision on at least a slight degree of disability), which largely coincides with the limitation or withdrawal of work for the respondents (9). Although we can suspect, that the premise for the efforts to get a status of disabled person to a greater extent may be a result of the overall situation on the labor market and the fear of being excluded from it due to age, than a real occurrence of disability, in the light of the findings of cited research on economic activity, it seems to be reasonable, that the proposed solutions in the field of retirement insurance take into account the projections of healthy life years expectancy, and in particular – the determinants of them functioning in the workplace.

### The Incidence of Occupational Diseases and the Risk of Accidents at Work in Different Occupational Groups in Poland

In all assessments made in the context of occupational diseases, two basic variables are always taken into account: sex and profession. As for the sex, much more often occupational illnesses are being predicated in case of men (10). This phenomenon is explained by the fact that, despite the ongoing sociocultural changes, sex continues to be strongly correlated with the type of work performed, and, thus, with greater exposure to specific health risks associated with the performance of a particular type of employment. Meanwhile, performing a particular job or employment in the selected sectors of the economy is exactly a key factor in exposure to occupational diseases. Among workers, who are characterized by the highest risk of incidence of occupational diseases, there are industrial workers, craftsmen, operators, and assemblers of machines and devices. What may seem surprising, the large number of occupational diseases has been recorded among specialists. This is probably caused by the fact that this group include teachers, among whom the prevalence of occupational diseases is relatively high (11).

Taking into account specific sectors of the economy and the absolute number of adjudicated occupational diseases, the industries with particular exposure to occupational illnesses include:

- agriculture, forestry, hunting, and fishing,
- industrial processing (especially the production of other non-metallic mineral and metal production),
- construction works,
- health care,
- education,
- mining and quarrying (especially, coal and lignite) (11).

A very important factor in terms of exposure to the occurrence of occupational disease is also age and experience of the employee. According to data of 2012, up to 92.6% of occupational diseases cases (except for allergies and contagious or parasitic infections, for which the exposure period is not taken into account) appeared after 10 years of exposure to harmful factors and 78% – after 20 years of long-term exposure (12). Working experience is also reflected in the age of people diagnosed with occupational diseases. As many as 84.5% of cases were diagnosed in persons over 45 years (12).

The trends are similar to statistics on accidents at work, to which the most vulnerable are two age groups. The first is a group of young employees between 21 and 34 years (due to lack of experience and short length of service), while the second, similarly as in the case of occupational diseases, are employees aged 45+ years (13).

### The Costs of Occupational Diseases and Accidents at Work

As shown above, age is correlated to the exposure on the incidence of occupational diseases and accidents at work. Particularly at risk are people aged over 45 years. In case such employees are affected by an occupational disease or are injured in accident, they not only do not generate income but also are subject to additional costs. At the level of European Union, the cost is estimated to vary between 2.6 and 3.8% of gross domestic product (14). It can be, therefore, indirectly concluded that this is the cost of years of healthy life lost due to improper working conditions – both in the context of mandatory insurance and the possibility of prevention, as well as the experience required for the acquisition of preferential pension rights. These costs in reality take, however, many dimensions.

The first is the economic dimension in the macro scale. These are mainly the costs of lost production in the national economy. The calculations show that the annual costs associated with lost production sold, due to time of incapacity for work, amounts to PLN 1 448 209 392. Lost gross value added for the same reason stands at 1.19% of GDP (439,958,512.00 PLN per year in 2004) (14).

Another cost at the macro level is the costs of social insurance benefits. This includes:

- disability benefits for accidents at work and occupational diseases (PLN 5 111.2 million),
- accident damages caused by diseases and accidents at work (PLN 225.7 million),
- accident benefits for permanent or long-term health damage or death due to an accident or occupational disease (PLN 1,822.5 million),
- benefits, allowances, and compensations for accidents at work and occupational diseases of social insurance:
  ○ compensation for accidents at work and occupational diseases,
  ○ compensation payments arising from the transfer to another job due to accidents at work or occupational diseases,
  ○ compensatory benefits, due to vocational rehabilitation.
- survivor’s benefits of accidents at work and occupational diseases (PLN 390,977,000).

These costs should be increased, yet, by the costs incurred by the employer, which include: benefits paid by the employer [the cost of damages, sickness absence, indemnities, and compensation allowances – a total of PLN 1.67 million in the year 2002 (14)], benefits for work in hazardous and burdensome conditions, and the costs of preventive measures.

Total expenditure are complemented by health-care costs and the costs spent by workers and their families.

In light of the above, it should be noted that longer professional activity of selected professional groups is correlated with an increased risk of occupational disease or accident at work. On the one hand, therefore, they are being kept in the labor market, in order to contribute to the development of the national economy by generating income, and on the other hand – such persons, by continuing their work, expose the social security system to expense. It should be emphasized that it is not LE, but healthy life years are the period of professional productivity, as only employee in good health contributes to economic growth.

## The Validity of the Use of Health Life Years Indicator in the Analyses in the Area of Public Health

Health life years (HLY) indicator is used in numerous projects and programs related to public health. It has been recommended as adequate for analysis in the area of public health by the resolution taken during the meeting of the Council of The European Union in Lisbon, March 23–24, 2000 (15). Nonetheless, it has never been used in the process of determining the age of retirement or the upper limit of productivity in specific occupations.

The Lisbon Strategy identified priority strategic objectives for policy decisions and structural reforms in Europe in the coming years. It focused on the social and economic welfare of the population, referred to in terms of productivity and active participation in social and professional life. It reoriented the economic life, which is to be based on the knowledge-based economy, assuring an increase in living standards. As a source of knowledge, the Lisbon Strategy indicates research at national and European levels. The reliable results of research in the area of public health shall contribute to economic growth and sustainable development (1).

Three years later, the Directorate for Health and Consumer Affairs of the European Commission, in cooperation with Eurostat, launched the concept of health monitoring in Europe [European Health Expectancy Monitoring Unit (EHEMU)]. Among its tasks, EHEMU listed, e.g., to improve health monitoring or collecting and collating data on the measurement of health status in the European Union. There were also two tasks dedicated to HLY: a control of the quality of calculation of this indicator and the development of training methods to facilitate the calculation (16).

The introduction of standardized instructions for HLY calculation is fully justified, because the indicator is calculated based on two variables: arrays of mortality (an objective statistical variable) and a standardized questionnaire concerning the measurement of disability included in the Eurostat surveys (Statistics on Income and Living Conditions Survey – EU-SILC), which is a subjective variable. Setting values, especially of the second variable, is burdened with a number of methodological difficulties (17). While the death (first variable) is an indisputable event, the occurrence of various health problems is seen differently by individual respondents. In the literature, there are over 300 different tools for evaluating health outcomes other than death (4). At the same time, we should take into account the fact that the HLY indicator was developed by Sullivan in the 70s of the last century (18). Since that time, much has changed, not only in terms of extending life or the level of mortality but also in the structure of diseases. Currently, the question of disability appears to be insufficient. It is also important to take into account chronic diseases or self-assessed health, as is the case of the modern methodological tool – a personal questionnaire based on the GALI (Global Activity Limitation Indicator) study (19).

Health life years indicator is also included in the health indicators of the European Union [European Community Heath Indicators (ECHI)] in the “Public Health Statistics” working group. This program was a component of the project of health monitoring and public health program for years 2003–2008, and its aim was to select indicators of public health, which would correlate with specified definitions of health and determinants of health (1). Moreover, the Joint Action on Healthy Life Years (JA: EHLEIS) is a joint action of European Member States (MS) and the European Union aiming at analyzing trends, patterns and differences in HLY, as well as in other summary measures of population health (SMPH) indicators, across the European MS (20).

European Community Heath Indicators work resulted in the development of the International Compendium of Health Indicators (ICHI), which allows them to be quickly defined and compared. In this compendium, measures relating to the expected state of health are included, for example HALE and Disability Adjusted Life Expectancy (DALE), but it lacked the HLY indicator. European Union defines the length of healthy life only by the measures used by Eurostat.

### Indicators Combining the Desired Health Status and Life Expectancy. Overview and Methodological Differences

A plurality of indicators of expected healthy life enables many ways of using them, but it is important that due to different algorithms for estimation and calculation, they cannot be used interchangeably, and, thus, the individual indicators are for specific uses. Below are listed and briefly discussed the most popular indicators.

- LE – the number of years of life measured on the basis of mortality in a given statistical year. It is a widely used indicator, but, on its basis, it is not possible to draw conclusions on the quality of life.
- Disability adjusted life years (DALY) – it indicates the number of years of potential life lost due to premature mortality and the years of productive life lost due to disability. The basic problem in calculating this indicator is the lack of reliable data. In result, there is often a need to use estimates, which substantially reduces the value and appropriateness of use the DALY indicator.
- DALE – to calculate DALE, out of the LE, the number of years with occurrence of any disability is subtracted. In 2001, this indicator was replaced with HALE indicator.
- HALE – it is the number of years that an average person in the population most likely will live in good health. It is important that HALE shows survival in different health states (summed), and not only in good health. Accordingly, it is characterized by high variability. It is being defined for men and women at the age of 0 and 65 years. The method of calculating HALE is more advanced than the method of calculating HLY and, therefore, it is difficult to collect complete data. HALE draws attention to the specific nature and severity of the disease, but often there are data gaps in the field of morbidity and mortality, preventing accurate calculation of the index.
- Potential years of life lost (PYLL) – is the number of years lost in the moment of premature death. This indicator is based on the assumption that the purpose of medical action is to save lives, prevent post-illnes complications, and if the disease has not occurred, it is to prevent it through prevention or early detection.
- Quality adjusted life years (QALY) – the oldest indicator of the quality of life in the context of health, a specific way of measuring the results of treatment. It combines data on mortality and quality of life in terms of health. It carries information about the impact of the disease from the perspective of the daily functioning of the patient and treatment assessment. It is an index based on a subjective evaluation test.

In view of the foregoing, it should be emphasized that HLY indicator is the most appropriate to be used in the economic analysis. Being relatively simple and, at the same time – in compliance with methodology proposed by the European Union, it makes possible to determine LE of healthy individuals and, therefore, a period without disability or limitations in productivity. At the same time, it is not fully objective measure, because the indicator is also based partly on a subjective survey.

## Conclusion

The matter presented in this article consists of a number of key issues. First – the complexity of the structure of social security systems has been highlighted, where solutions the system consist of are the result of several categories of factors: a traditional set of design principles referring to the protection against social risks associated with the loss of earning capacity; political processes associated with the efforts of certain groups to secure greatest possible range of own social security, but also the processes of competition for political power, in which decision makers are trying to secure themselves an electoral support; and finally the socioeconomic factors, which have an impact on the possibility of securing financial foundations for the level of state responsibility in the social sphere, which has been promised to the public, but which are also associated with the need to secure an appropriate level of supply in the labor market in light of the observable demographic transitions.

In light of the observations of decision-making processes that shape social security system in Poland in recent years, a deficit of discourse referring to reliable data in the area of public health should be noted, that would reflect epidemiological conditions of the functioning of the labor market, in particular – in given segments of this market. As a result, one can meet cases of justifying changes to the retirement insurance with the statistical data on lengthening the LE or on differences in this regard between men and women, but definitely it lacks the in-depth analysis of this issue, that would take into account specific data showing differences in the level of health risk characterizing various professional groups. Exceptions to the generally applicable rules of acquiring pension rights seem to be still, and to a greater extent, dependent on factors of a purely political nature. In result, there is a risk that the social security system, in particular retirement system, becomes inadequate to the actual social needs, differentiating the status of individuals in an unfair way, and not based on real epidemiological and economic evidence. On the other hand, there is a danger that the general trend to extend professional activity, not accompanied by appropriate actions to secure the health status of employees, will give the illusory only savings, while generating increased costs in other segments of the social security system, as health care or disability protection system, in addition to not guarantee expected increase in revenues from contributions, as a result of declining productivity of workers affected by health problems resulting from the specific conditions or the way of performing their work.

Therefore, there is a need to formulate the tools that will become a foundation for defining principles of functioning of the social security systems, and which will correspond to the real needs of society, and in the most precise way will define the health-related subsoil for rights under a retirement scheme, at the same time providing data to estimate the cost effectiveness of various solutions in this regard. The HLY indicator discussed in the article, due to its relative simplicity, high informational value, and, finally, its compliance with the guidelines elaborated by the European Union, presents itself as a tool to meet these requirements, and, as such, it should be implemented in designing social security solutions for different professional groups.

## Author Contributions

PR conceived the study, outlined the general message and theses, and prepared final version of the paper. KB collected the necessary data and prepared the draft version of the paper.

## Conflict of Interest Statement

The authors declare that the research was conducted in the absence of any commercial or financial relationships that could be construed as a potential conflict of interest.
